# Metal Ion Enhanced Charge Transfer in a Terpyridine-bis-Pyrene System

**DOI:** 10.3390/s90503604

**Published:** 2009-05-13

**Authors:** Anthony D'Aléo, Elio Cecchetto, Luisa De Cola, René M. Williams

**Affiliations:** 1 Molecular Photonics Group, Van't Hoff Institute for Molecular Sciences, Universiteit Van Amsterdam, Nieuwe Achtergracht 129, 1018 WS Amsterdam, The Netherlands; E-Mail: anthony.daleo@gmail.com; 2 Physikalisches Institut, Westfälische Wilhelms-Universität Münster, Mendelstrasse 7, D-48149 Münster, Germany; E-Mail: decola@uni-muenster.de

**Keywords:** branched molecules, spectroscopy, ion complexation, excited state absorption, emission, molecular polarity sensor

## Abstract

The synthesis, electrochemical and photophysical properties of a branched molecule 3,5-bis(pyrene-1-yl)-4′-phenyl-2,2′:6′,2″-terpyridine are reported. Spectroscopy in different solvents reveals that an optical electron transfer from the pyrene donor to the terpyridyl electron acceptor can occur in polar media, as the system displays both charge transfer (CT) absorption and CT emission. Furthermore, the study of the zinc complex as well as the bis-protonated form shows an enhancement of the electron transfer character of the system, by an increase of the acceptor strength. This is accompanied by a large increase of the non-radiative processes. With sub-nanosecond transient absorption spectroscopy, the CT state, consisting of the pyrene radical cation and the terpyridine radical anion, has been detected. At room temperature, the study of the nanosecond transient absorption spectra reveals the formation of a low-lying triplet excited state that we attribute to the pyrene moiety through which the CT state decays. At 77K, the absence of the terpyridine triplet emission also suggests the population of a low-lying triplet state of the pyrene unit.

## Introduction

In the last decades, in order to gain more insight into electron transfer processes, extensive studies have been carried out on the optical behavior of chromophoric molecular systems consisting of electron donor and acceptor groups connected via different bridges [1,2]. Because of its long fluorescence lifetime (up to 450 ns) [3], high fluorescence quantum yield [4] and its ability to act as a donor [5-9] as well as acceptor [9-12], pyrene has often been chosen as an ideal charge transfer partner. In some cases, the pyrene moiety was attached to chelating “polypyridyl” systems, which are known to coordinate d^7^ to d^10^ metal-ions, and through this specific metal ion complexation the excited state properties can be tuned. As conjugated spacers between the pyrene and the metal centers, phenylene or ethynylene-phenylene have been reported. Among the innocent metal ions able to coordinate bipyridine units, Zn(II) has received attention because of its ability to enhance the emission of the ligands and tune some of the properties. For example, in the poly- and oligo-phenylene vinylene (OPV) derivatives covalently linked to a bipyridine system reported by Wasielewski *et al.* [13] an enhancement of the electronic delocalization on the polymer backbone and a red shift of the emission was observed upon addition of particular ions [e.g. Zn(II)]. The emissive excited state was suggested to have charge transfer character. More recently, systems have been used in order to show the occurrence of emissive charge-transfer states in their zinc (II) complexes, such as bipyridine linked to a donor group [14] or to a pyrene unit via an oligo-phenylene bridge [15,16], conjugated pyrene-thiophene-terpyridine [17], or OPV terminated by a terpyridine unit. [18] Also in free ligands such as bipyridine bound to pyrene [19] and terpyridine linked in its 4′-position to a dimethylaniline group [20] charge transfer emission was observed.

The special interest in the terpyridine derivative systems lies in e.g. the strong blue emission of their zinc complexes. Furthermore, the zinc can be used to assemble different units and build up long rod-like linear structures which can be considered coordination polymers. [21,22] Due to the dynamic nature of the systems and to the high emission quantum yield in the blue region, zinc bis(phenylterpyridine) derivatives [23,24] have attracted the attention in the material-science field. Two recent reviews on polypyridyl systems in conjunction with aromatic units [25] and with respect to molecular wires [26] are available.

**Figure 1.**
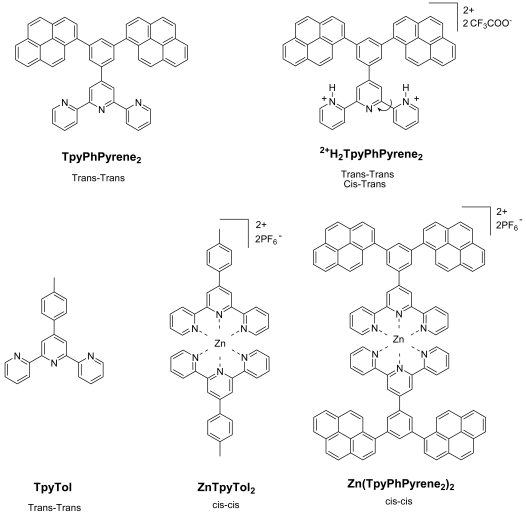
Structures and abbreviations of the molecules synthesized and investigated. Preferred conformation is also indicated.

In order to design systems where the emission can be tuned and to have a full understanding of the free ligand and its behavior once coordinated to zinc, we have investigated the properties of a pyrene substituted terpyridine, its zinc complex and the bis-protonated form of the ligand. The synthesis, electrochemical behavior and the photophysics of these species composed of a terpyridine (Tpy) ligand which is linked to a phenyl substituted with two pyrene units in meta positions (Figure 1) are described.

The term dendrimer, a contraction of the Greek words *dendros* for “tree” and *meros* for “part”, can be applied to branched, tree like molecules. **Zn(TpyPhPyrene_2_)_2_** has four pyrene branches and a **Zn(Tpy)_2_** core and as such can be considered a first generation dendrimer. Expansion of the dendrimer into higher generations can in principle be attained by functionalization of each pyrene with, e.g. a 3,5-bis(pyrene-1-yl)-phenyl unit at the 8-position. **Zn(TpyPhPyrene_2_)_2_** is a branched molecule and a simple (first generation) model for such highly branched fluorescent dendrimers, but it is also a dendritic polarity probe. As will be shown, **Zn(TpyPhPyrene_2_)_2_** is very sensitive towards polar solvent molecules and as such can be described as a fluorescent dendritic sensor for the polarity of its environment.

## Results and Discussion

### Synthesis

**Scheme 1.**
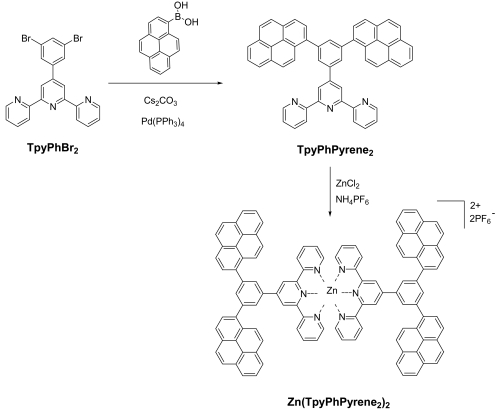
Synthesis of the bispyrene terpyridine ligand and its zinc complex.

The di-halogenated terpyridine (**TpyPhBr_2_**) was prepared by using a slight modification of a literature method [27] (see Experimental section). The free ligand **TpyPhPyrene_2_** was synthesized by coupling **TpyPhBr_2_** with 1-pyreneboronic acid following the Suzuki cross coupling procedure (Scheme 1) [28,29].

The zinc complex was prepared by refluxing **TpyPhPyrene_2_** with ZnCl_2_ in methanol followed by precipitation of the hexafluorophosphate salt by addition of ammonium hexafluorophosphate, as reported before (see Scheme 1) [30].

The bis-protonated molecule was prepared *in situ* prior to measurement by adding 100 μL of a 1 M trifluoroacetic acid solution (in the solvent required) to a 2 mL solution of **TpyPhPyrene_2_** (Abs ≈ 0.2).

The ligand **TpyPhPyrene_2_** and the zinc complex **Zn(TpyPhPyrene_2_)_2_** were isolated and fully characterized by ^1^H-NMR, ^13^C-NMR and High Resolution Electrospray Ionization mass spectrometry.

### Electrochemistry

The study of the electrochemical properties of the ligand **TpyPhPyrene_2_** was performed in deaerated THF (vs SCE). Two irreversible cathodic peaks at -1.57 V (small hump) and -1.91 V (involving at least two electrons) are observed by cyclic voltammetry. On the basis of the comparison to literature data [31,32], we attribute the lowest reduction process to the terpyridine reduction in which the central pyridine is reduced first around -1.57 V and the pyrenes reducing at higher voltage (-1.9 V). The oxidation process that occurs at E_p,a_= +1.36 V is also irreversible. This is consistent with the oxidation of the pyrene, which occurs at this potential in acetonitrile [33]. In this case, the lack of reversibility in the redox behavior is suggested to arise because of the formation of a dimer by π-π interaction of one radical cation of pyrene with a second neutral pyrene molecule [34].

For **Zn(TpyPhPyrene_2_)_2_**, no metal centered redox processes are expected for the d^10^ zinc ion and none were observed. In this case, the same irreversible redox processes were observed without a significant shift.

As **TpyPhPyrene_2_** contains an electron donor (pyrene) and an electron acceptor (Tpy), it can display charge transfer characteristics. From the electrochemical data, it is possible to estimate the thermodynamic energy change for electron transfer (Gibbs free energy change: ΔGeT) in such a system by using a Weller-type analysis [35]. The E_00_ can be estimated using the maximum emission of the donor at 77 K in butyronitrile matrix (3.28 eV, see luminescence part). Neglecting the work term, it is found that the ΔG_eT_ is negative in the solvent in which the electrochemistry was performed, THF, (E_ox_ - E_red_ - E_00_ = 1.36 + 1.56 - 3.28 = -0.36 V). As the redox processes are irreversible, the value obtained is a rough estimate. This negative value shows that the process is thermodynamically allowed. The increase of the solvent polarity, relative to THF, will result in a more negative ΔG_eT_.

### UV/Visible absorption

**Figure 2.**
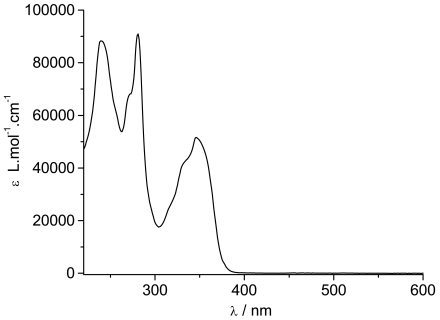
UV/Visible absorption spectrum of **TpyPhPyrene_2_** in THF.

The ground state absorption spectrum of **TpyPhPyrene_2_** is shown in Figure 2. It shows three absorption bands centered at 240 nm, 280 nm and 347 nm with two shoulders at 270 nm and 332 nm. The spectra obtained in low polarity solvents (e.g. toluene, tetrahydrofuran, dichloromethane) and in high polarity (e.g. acetonitrile, dimethylformamide) environment are identical, within experimental error.

By comparing the spectrum with those of 1-phenylpyrene [10,36] and of substituted phenyl-terpyridine [20], all bands can be identified. The 240 nm band, the shoulder at 270 nm and the 347 nm bands belong to the allowed π-π* transitions of the phenylpyrene moiety while the band at 280 nm is attributed to π-π* transitions of the Tpy unit. The second shoulder at 332 nm seems to be due to a contribution from both parts. Apart from the Tpy bands, the spectrum consists of the characteristic absorption bands of substituted pyrene where the typical vibronic structure of pyrene is broadened by the interaction between the π-electron systems of the pyrene (or alkylpyrene [37,38]) and the phenyl units [19].

The molar absorption coefficients are quite high for each band (see Table 1) revealing allowed π-π* transitions (Table 1). If a weak charge transfer band should be observed, we are expecting it in the UV region (around 340 nm) as also reported elsewhere [23,39].

**Figure 3.**
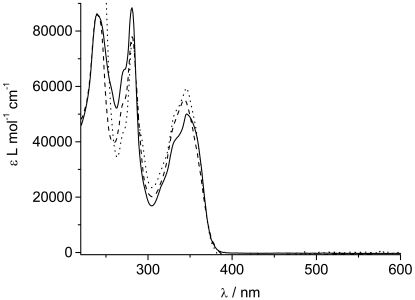
UV/Visible absorption spectra of **TpyPhPyrene_2_** (—), **Zn(TpyPhPyrene_2_)_2_** (divided by 2)(---) and **^2+^H_2_TpyPhPyrene_2_** (⋯) in acetonitrile.

**Figure 4.**
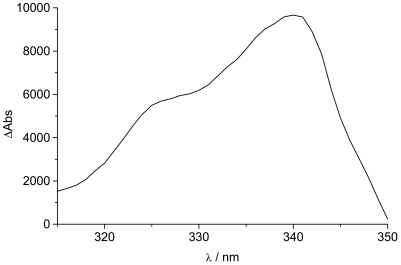
Differential spectrum obtained by subtracting the UV/Visible absorption spectrum of **TpyPhPyrene_2_** from the one of **Zn(TpyPhPyrene_2_**)**_2_** (divided by 2) in THF.

The UV/Visible spectrum of the zinc complex **Zn**(**TpyPhPyrene_2_**)**_2_** is almost super-imposable over that of the free ligand **TpyPhPyrene_2_** (Figure 3). Only the lowest energy transition is slightly blue shifted upon addition of the zinc(II) (from 347 nm to 342 nm). This shift, accompanied by an increase of the molar absorption coefficient, can be attributed to zinc complexes where a new band, growing around 330 nm, is reported. This band is attributed to the cis-cis form for other zinc(II) terpyridine complexes where the nitrogen complexation induces the ligand to convert from the trans-trans to cis-cis form [40,41]. Moreover, the increase in the molar absorption coefficient of the 342 nm band is accompanied by a slight decrease of the 281 nm band (from 88,400 L mol^-1^ cm^-1^ to 78,000 L mol^-1^ cm^-1^; Table 1). This increase of the molar absorption coefficient can be attributed to an Intramolecular Ligand Charge Transfer (^1^ILCT) transition, which can appear as a shoulder at 340 nm [23,39]. In order to identify the ILCT absorption band, a differential spectrum was obtained by subtracting the UV/visible absorption spectrum of **TpyPhPyrene_2_** from the one of **Zn**(**TpyPhpyrene_2_**)**_2_** (divided by 2) both in THF solution (see Figure 4).

A band at 340 nm can be observed, which we assign to an ^1^ILCT transition. The appearance of such a band (with a relatively high extinction coefficient) suggests that upon complexation with the zinc ion, the electron withdrawing character of the Tpy moiety increases and therefore, the donor acceptor interaction becomes stronger. The ^1^ILCT transition appears to borrow intensity from the pyrene-Tpy based transitions which results in the enhancement of the charge transfer absorption band that is overlapping with the pyrene bands [42-46]. The structure in the CT absorption band can be due to the presence of two conformations or to a slight spectral shift as compared to the reference systems.

Upon addition of trifluoroacetic acid to the solution of **TpyPhPyrene_2_**, a bis-protonation is expected [23]. The absorption spectrum is almost identical to that of the zinc complex (Figure 3). The main difference is a negligible blue shift of the lowest energy band compared to the free ligand (346 nm for **^2+^H_2_TpyPhPyrene_2_**; 347 nm for **TpyPhPyrene_2_**) which shows that upon protonation, conformational changes are not the same as upon zinc complex formation. We believe, in fact, that only one of the pyridine rings changes conformation from trans to cis in order to “coordinate” one proton with the central pyridine [47]. The molar absorption coefficient of the 347 nm band of **^2+^H_2_TpyPhPyrene_2_**, (Table 1) is higher than that of **TpyPhPyrene_2_** and **Zn**(**TpyPhPyrene_2_**)**_2_**, which indicates that the ^1^ILCT absorption can be observed here as well and that this transition is even more favorable than for **Zn**(**TpyPhPyrene_2_**)**_2_**.

### Luminescence study

**Figure 5.**
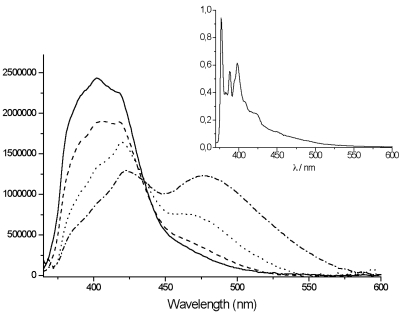
Emission spectra of **TpyPhPyrene_2_** in THF (—), in valeronitrile (---), in propionitrile (⋯) and in acetonitrile (–·–) at room temperature (inset: spectrum at 77K in butyronitrile matrix) (λ_ex_= 310 nm).

The fluorescence emission spectra of **TpyPhPyrene_2_** and **Zn**(**TpyPhPyrene_2_**)**_2_** were found to be independent on the excitation wavelength for all solvents studied. Interestingly, the shape of the emission bands and the emission maxima depend strongly on the solvent used (Figure 5). These phenomena are attributed to a solvatochromic shift of the emission maximum as often observed for charge transfer transitions [19,48-50]. The shape of the emission bands is independent of the sample concentration (in the 10^-4^ to 10^-8^ M range), thus ruling out intermolecular excimer formation.

The emission spectrum of the free ligand in THF (Figure 5) shows a broad but somewhat structured emission band centered at 400 nm that is attributed to the local excited (LE) state of the pyrene. However, in nitrile solvents the spectra show a dual emission with a second band arising around 470 to 480 nm (with increasing polarity).

The appearance of the low energy emission band for the **TpyPhPyrene_2_** ligand in highly polar solvents implies the existence of a low lying excited state polar in nature and stabilized by the solvent that is due to a charge separation between the electron donor (pyrene) and the electron acceptor (terpyridine) moieties of the molecule.

**Figure 6.**
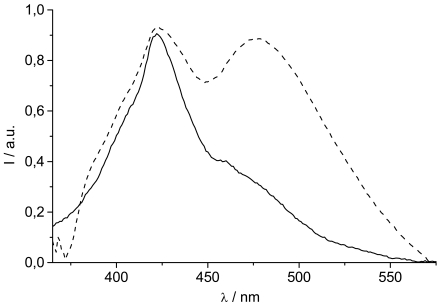
Comparison the emission spectra of **TpyTol** in ethanol (—) and **TpyPhPyrene_2_** (---) in acetonitrile (λ_ex_= 310 nm).

Upon closer examination of Figure 5, it can be seen that the so called local excited state emission also changes shape with polarity. Whereas the spectra in THF and valeronitrile have a typical shape that can be attributed to “substituted pyrene”, the spectra in propionitrile and acetonitrile clearly have a different shape. This is in contrast with a π–π* transition localized on the pyrene (Ham effect: increase of 0-0 transition with polarity). In fact, these spectral features are very similar to tolyl-terpyridine (**TpyTol)** emission in polar solvent. It thus appears that several close lying excited states are present and a reordering of states occurs upon solvent polarity change. A comparison of the emission spectrum of **TpyPhPyrene_2_** with that of **TpyTol** is given in Figure 6.

Clearly, a simple picture with only one LE state is not fully correct and a manifold of two or more states must be considered as fluorescent levels. Of course, it is not possible to fully rule out the possibility that the 425 nm band is just an enhancement of the vibronic structure of the pyrene (or 1,3-dipyrenyl-benzene) that gains intensity vs the other vibration levels due to the change in solvent.

The excited state lifetimes were determined in a number of solvents (see table 2). The LE state displays a lifetime between 17 and 20 ns in most non-polar solvents. In polar media, a bi-exponential decay of the emission can be observed due to the formation of a lower lying ^1^ILCT state (Figure 5) that has a shorter lifetime (approximately 6 ns, Table 2) and a lower energy maximum with increasing the polarity of the solvent.

The luminescence quantum yield of the overall emission (LE + ILCT) for **TpyPhPyrene_2_** varies between 12 % and 29 % (see Table 2). The population of the CT state can be modulated by changing the polarity in a series of nitrile solvents (Table 3). The increase of the amplitude of the 6 ns component with increasing the polarity reveals a more efficient transfer to the ^1^ILCT. In the same table, we have collected the different excited state lifetimes for the two emissions (LE, τ_1_ and ILCT, τ_2_) as well as the different weights (Amplitude τ). We have analyzed the behavior in a series of nitrile solvent where only the polarity increases going from butyronitrile to acetonitrile. All the spectra have been repeated in deaerated solutions but no influence of oxygen was observed.

**Figure 7.**
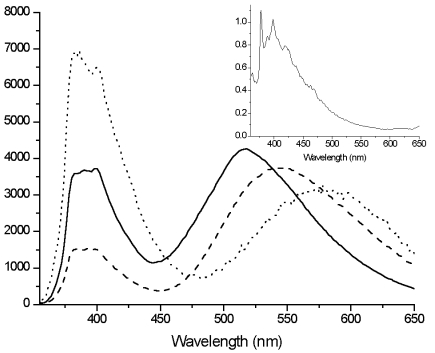
Normalized emission spectra of **Zn(TpyPhPyrene_2_)_2_** in THF (—), in dichloromethane (---) and in acetone (⋯) at room temperature (inset: spectrum at 77K in butyronitrile matrix)(λ_ex_= 310 nm).

For **Zn**(**TpyPhPyrene_2_**)**_2_**, two bands can be observed in the emission spectra (Figure 7): a sharp local emission band and a broad structureless charge transfer emission band. The high energy band that is slightly structured can be attributed either to the pyrene or to the Tpy perturbed by the zinc ion.

The lowest energy band observed at 518 nm (in THF) to 616 nm (in acetonitrile) is strongly solvatochromic and is red shifted in all solvents, as compared to the **TpyPhPyrene_2_** emission (Table 4). Such a solvent dependence is again incompatible with localized π-π* emission from either the Tpy or the pyrene moiety, but it is consistent with emission from a ^1^ILCT state. This state emits at lower energy when the zinc is coordinated to **TpyPhPyrene_2_** due to the ionic charges introduced by the zinc which makes the Tpy a stronger electron withdrawing group. A strong decrease of the quantum yield of emission was observed as compared to that of the free ligand (cf. Tables 2 and 4). The quantum yields measured for **Zn(TpyPhPyrene_2_)_2_** show a slight variation: from more than 5% in a nonpolar solvent to less than 0.3% in polar media. The emission intensity drops strongly when passing from dichloromethane (Δƒ = 0.218) to acetone (Δƒ = 0.284).

The emission centered around 400 nm shows a mono-exponential decay on the order of 1 to 2 nanoseconds in every solvent (much shorter than that of the free ligand). The emission lifetime on the red side of the spectrum, which is attributed to the ^1^ILCT shows a lifetime between 3 and 14 ns. It is interesting to note that for **Zn(TpyPhPyrene_2_)_2_**, the ^1^ILCT lifetime is decreasing when going to more polar solvents which is in agreement with the decrease in the energy gap between the excited state and the ground state (energy gap law).

The bis-protonated terpyridine **^2+^H_2_TpyPhPyrene_2_** in chlorinated solvents shows the same emission bands as the zinc complex. For this system, the LE state emission can be estimated to be reduced more than 500 times and the ^1^ILCT emission is more red-shifted. Clearly, protonation or coordination of the nitrogen atoms of the **TpyPhPyrene_2_** strongly increases the non-radiative processes in this system.

In rigid matrices at low temperature (77 K), the fluorescence emission spectra of all compounds show emission bands with vibronic structure and no structureless CT-emission band can be observed (see inset Figures 5 and 7) indicating that the intramolecular electron transfer does not occur at low temperature, which corroborates the room temperature assignments. The long excited state lifetimes at 77 K (89 ns for **TpyPhPyrene_2_** and 21 ns for **Zn(TpyPhPyrene_2_)_2_**) also indicate that the structured fluorescence is a pyrene-based emission since the corresponding Tpy emission decays in less than 1 ns. At this temperature, even for the zinc complex, no emission coming from the Tpy triplet state can be observed as for other Tpy derivatives. This suggests that the triplet state of the Tpy is quenched by a very fast triplet-triplet energy transfer from the Tpy state to the lowest ^3^Py* state located around 640 nm [51] which is apparently non-emissive at 77 K in this case.

### Transient Absorption spectroscopy

**Figure 8.**
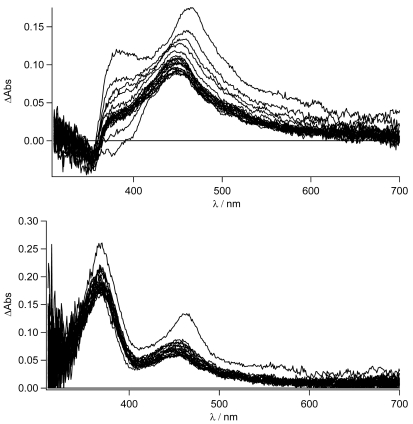
Nanosecond transient absorption spectra of **TpyPhPyrene_2_** (top) and of **Zn**(**TpyPhPyrene_2_)_2_** (bottom) in acetonitrile with 5 ns incremental time delay (λ_ex_= 290 nm).

**Figure 9.**
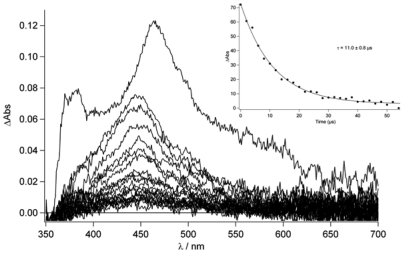
Nanosecond transient absorption spectrum for **TpyPhPyrene_2_** in deaerated acetonitrile with 2 μs incremental time delay (λ_ex_= 290 nm). The kinetics of the triplet state decay is depicted in the inset.

**Figure 10.**
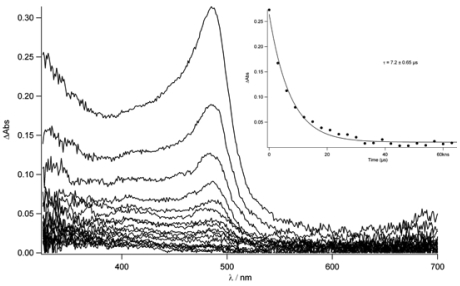
Nanosecond transient absorption spectrum of **TpyTol** in acetonitrile with λ_ex_= 290 nm and 2 μs increment.

To gain a better insight into the processes in and the nature of the excited states of **TpyPhPyrene_2_** and of **Zn(TpyPhPyrene_2_)_2_** at room temperature, time resolved absorption spectroscopy was employed. Spectra on the nanosecond time scale are depicted in Figures 8 to 10.

In Figure 8, the transient absorption spectra of **TpyPhPyrene_2_** and of **Zn(TpyPhPyrene_2_)_2_** in CH_3_CN are depicted. Both spectra are characterized by two positive absorption bands at 463 nm and at 380 nm which can be attributed to the radical cation of the pyrene (463 nm) and the radical anion of the terpyridine (380 nm). For both compounds, the charge separated state decays bi-exponential on a 6 and ∼12 ns timescale and is converted into a blue shifted absorption band with a long lifetime. The bi-exponential decay indicates excited state equilibration. The long-lived species has a maximum absorption at 450 nm and can be assigned to a triplet-triplet absorption (see Figure 8).

It is interesting to notice that, as for the emission spectroscopy, in CH_3_CN **TpyPhPyrene_2_** has a longer lived CT state (τ_em_ = 6-8 ns, see Table 3) as compared to that of the zinc complex (τ_em_ = 3 ns, see Table 4). In the transient spectra, a similar kinetic behavior is observed. The decay of the triplet state can be followed better in Figure 9, which shows similar spectra as in Figure 8 in deaerated conditions and with 2 μs incremental time delay. As it can be seen, the lifetime of this excited state is ca. 11.0 μs (see inset Figure 9) while, in aerated condition, a lifetime of 240 ns is found.

In order to establish if the triplet state belongs to the pyrene moiety or to the Tpy unit, the transient absorption spectrum of **TpyTol** in acetonitrile was recorded (Figure 10). In fact, this spectrum shows a sharp peak around 490 nm which decays within 530 ns in aerated conditions while, after degassing, a longer lifetime was found (7.2 μs). This absorption can clearly be attributed to the terpyridine triplet state (T_1_→T_n_).

On the basis of this experimental evidence and on the literature reports [52,53], we assigned the long lived transition observed in Figure 9 to the T_1_→T_n_ absorption of the pyrene moiety. In our systems, the T_1_→T_n_ is localized in the 430-460 nm region. It thus seems most likely that phenyl substitution of pyrene shifts its triplet-triplet absorption to slightly longer wavelengths than that reported for unsubstituted pyrene (410-430 nm).

**Figure 11.**
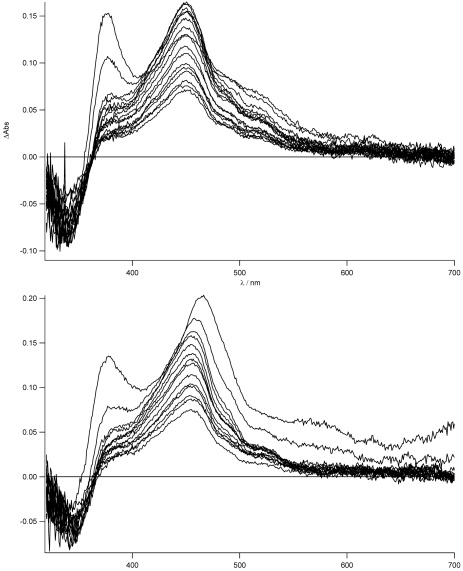
Top: Transient absorption transient spectra of **TpyPhPyrene_2_** in THF (increment 10 ns, λ_ex_= 300 nm); bottom: transient absorption transient spectra of **Zn(TpyPhPyrene_2_)_2_** in THF (increment 10 ns, λ_ex_= 300 nm).

To support the formation of the charge separated state and to prove that this effect is strongly dependent on the solvent and more important on the presence of the zinc, we have measured the transient absorption spectra in THF for **TpyPhPyrene_2_** and **Zn(TpyPhPyrene_2_)_2_**. The results are shown in Figure 11.

**Figure 12.**
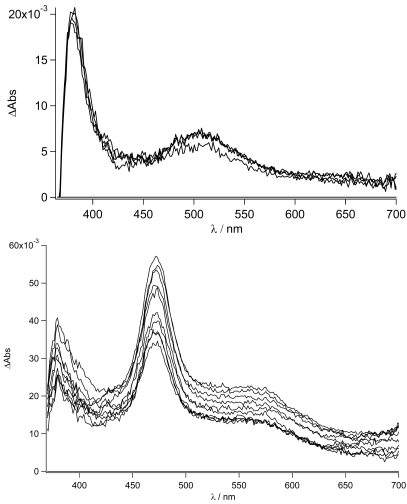
Top: Sub-nanosecond transient absorption spectra of **TpyPhPyrene_2_** in THF with λ_ex_= 340 nm and 200 ps incremental time delay, Bottom: Sub-nanosecond transient absorption spectrum of **TpyPhPyrene_2_** in acetonitrile with λ_ex_= 340 nm and 100 ps incremental time delay.

As can be easily seen for the free ligand in the nonpolar solvent, no evidence (lack of the 463 nm band) of the CT state is observed. The local singlet excited state, populated in the first nanoseconds, is visible (see also later) and transforms into the long lived triplet state. On the other hand, for the zinc complex in THF, the stronger charge transfer character of its excited state is mirrored in the formation of the charge separated state at 463 nm as was already reported in acetonitrile (see Figure 8). In this case, no indication of the population of the LE state is observed. The decay to the triplet state of the pyrene fits with the longer lived CT lifetime measured by time resolved emission spectroscopy (τ = 14 ns). The sub-nanosecond transient absorption spectra are shown in Figure 12 in THF and acetonitrile, respectively, for **TpyPhPyrene_2_**.

As expected from the nanosecond transient absorption spectra and in accordance with the emission spectra that were reported for **TpyPhPyrene_2_** (see Figure 5), the transient absorption spectra obtained in the two solvents are quite different. In the nonpolar medium THF, the only visible transitions are those due to S_1_→S_n_ of the Tpy, at high energy (390 nm), and those belonging to the pyrene units (in the 470-530 nm region) [54,55]. In the polar solvent (acetonitrile), the spectra present the singlet absorption of the Tpy (at 390 nm) but, at lower energy, two distinct features are present which cannot be attributed to the pyrene singlet absorption (Figure 12 bottom). In fact, as we have already discussed, a charge transfer in acetonitrile is thermodynamically allowed leading to the formation of the radical cation of the pyrene [56,57] and the radical anion of the Tpy clearly visible at 463 nm and 550 nm, respectively. This CT state decays on a nanosecond time scale as mentioned before. The formation of the CT state from the singlet excited state could not be detected, indicating direct charge transfer state formation by excitation in the CT absorption band (optical electron transfer). In our time regime (up to 1ns), no other states are populated and due to the long lifetime (τ = 17 ns), no clear decay is observed. It is interesting to notice that the band at 470 nm does not show a clearly monoexponential decay. This may be due to the formation of the pyrene triplet state that, unfortunately, overlaps with the CT absorption band.

**Figure 13.**
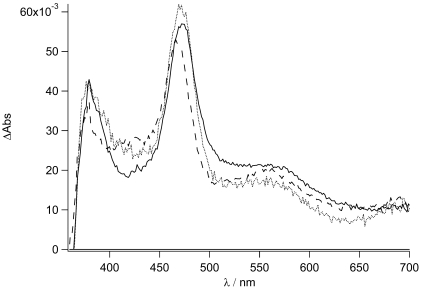
Sub-nanosecond transient absorption spectra of **TpyPhPyrene_2_** (—), **Zn(TpyPhPyrene_2_)_2_** (&ctdot;) and **^2+^H_2_TpyPhPyrene_2_** (---) in acetonitrile with λ_ex_= 340 nm, at 200 ps incremental time delay.

For the zinc complex and the bis-protonated species, the electron transfer is expected to be more exergonic and, indeed, the transient absorption spectra show the typical features of the formation of the CT state (see Figure 13).

## Conclusions

**Scheme 2.**
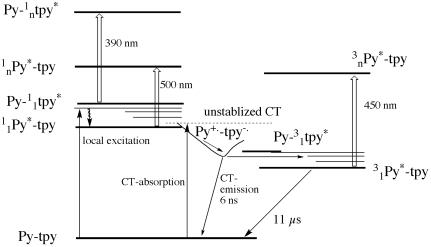
Schematic representation of the deactivation pathways. in presence of the electron transfer process, of Py-tpy (generalization of a pyrene-terpyridyl system). Excited state localization, absorption and multiplicity are also indicated: ^3^_1_Py indicates e.g. the first triplet state of Pyrene, with subscript n indicating higher lying states.

The properties of 3,5-bis(pyrene-1-yl)-4′-phenyl-2,2′:6′,2″-terpyridine, its bis-protonated form and its zinc complex were studied. The electrochemistry of **TpyPhPyrene_2_** in combination with a Weller-type analysis shows that photoinduced electron transfer process is thermodynamically allowed in polar solvents. The steady state and time resolved spectroscopy indicate the presence of a photoinduced electron transfer process for **Zn(TpyPhPyrene_2_)_2_**) in all solvents, and for **TpyPhPyrene_2_** only in polar solvents. A general deactivation pathway is proposed (Scheme 2). The systems displays a charge transfer absorption band, and charge transfer emission in many solvents.

As can be seen in Scheme 2, the un-relaxed charge transfer state to which direct absorption can occur is very close in energy to both the local excited state of the pyrene and of the terpyridine. The excited state absorption of the latter two excited states can be observed for the ligand in non-polar solvents. In polar solvent and for the zinc complex, we observe a population of a short lived charge transfer state that shows emission and that decays mainly to a triplet state localized on the pyrene unit. The transient absorption spectroscopy confirmed the charge separation phenomenon revealing typical bands of the radical cation of the pyrene and radical anion of the Tpy.

The modulation of the emission of metal complexes by substitution with chromophoric units is a delicate interplay of different excited states where the strongly emissive properties (of e.g. zinc-bis-terpyridyl) can be lost through charge transfer states with small quantum yields of emission and to dark local triplet states. Strongly emissive charge transfer states like observed for the free ligand **TpyPhPyrene_2_** are still relatively rare and difficult to design, but white light emission [58] (for e.g. OLED lightning purposes) is attainable.

The substitution of phenyl-terpyridines with electron donating pyrene groups can lead to the development of new charge transfer systems that have special emissive properties that can be modulated by complexation.

## Experimental

### General

All chemicals were purchased from Acros or Aldrich and were used as received. All solvents for synthesis were analytic grade. For the spectroscopy, spectroscopic grade solvents were used. ^1^H-NMR spectra were obtained with a Varian Gemini-300 spectrometer. Chemical shifts (δ) are given in ppm, using the deuterated solvent as internal standard.

### Synthesis of ***TpyPhBr*_2_**

To a solution of 3,5-bromobenzaldehyde (1 eq) in methanol (50 mL) and aqueous sodium hydroxide (1 mol/L, 1 eq) was added 2-acetylpyridine (1.05 eq) and the mixture was stirred for 3 hours. The precipitate was filtered off on a glass filter and recrystallised from ethanol. The precipitate (3,5-dibromoazachalchone) was dissolved in dichloromethane (100 mL) and was then washed with water and dried over MgSO_4_. The solvent was evaporated under vacuum and the solid was used without further purification.

In a round bottom flask, potassium tertbutoxide (2 eq) was solubilized in dry THF (100 mL) Then, acetylpyridine (1 eq) was added. The reaction was stirred for 4 hours (becoming slowly pink) at room temperature under nitrogen. 3,5- Dibromoazachalchone (1 eq) in dry THF (25 mL) was added. The reaction was stirred under nitrogen at room temperature for 4 hours (becoming quickly red). Then, THF was evaporated. Ammonium acetate (10 Eq) in ethanol (50 mL) was added and the reaction was stirred at 70 °C with the flask aerated during 36 hours. Then, the solvent (black solution) was removed. The solid was dispersed in water and filtered. This solid was intensively washed with water then solubilized in CHCl_3_ and dried over MgSO_4_. The product was recrystallized from ethanol (3 times). Yield: 43% (M= 467.17 g/mol); ^1^H-NMR (300 MHz, CDCl_3_): δ (ppm) = 8.76 ppm (d, ^3^*J*= 4.8 Hz, 2 H), 8.70 ppm (s, 2 H), 8.66ppm (s, 2 H), 7.97 ppm (d, ^4^*J*= 1.5 Hz, 2 H), 7.91 ppm (t, ^3^*J*= 8.1 Hz, 2 H), 7.76 ppm (d, ^4^*J*= 1.5 Hz, 1 H), 7.36 ppm (t, ^3^*J*= 4.8 Hz, 2 H).

### Synthesis of *TpyPhPyrene*_2_

In a 100 mL Schlenk flask, **TpyPhBr_2_** (100 mg, 0.214 mmol), pyrene-1-boronic acid (132 mg, 0.535 mmol) and cesium carbonate (418 mg, 1.284 mmol) were mixed in DMF (30 mL) and the solution was degassed. A catalytic amount of Pd(PPh_3_)_4_ was added (25 mg, 0.021 mmol). The reaction was heated during 60 hours at 100°C under nitrogen. The solution was filtered and the DMF was removed with toluene (azeotrope). The solid was washed with pentane and filtered off. The solid was solubilized in chloroform dried with MgSO_4_, filtered and evaporated under vaccum. The solid was purified by chromatography (alumina oxide) using a mixture dichloromethane/ethyl acetate (7:1). Yield: 35%, m = 54 mg (M = 709.86 g/mol); ESI-MS: 710.20; *1*H-NMR (300 MHz, CD_2_Cl_2_) (see below): δ (ppm) = 8.98 (s, 2 H), 8.70 (d, ^3^*J*= 7.8 Hz, 2 H), 8.66 (d, ^3^*J*= 5.4 Hz, 2 H), 8.45 (d, ^3^*J*= 9.0 Hz, 2 H), 8.35-8.00 (m, 19 H), 7.89 (dt, ^3^J= 7.2 Hz, ^4^*J*= 1.8 Hz, 2 H), 7.36 (t, ^3^*J*= 4.8 Hz, 2 H); ^13^C-NMR (125.7 MHz, CD_3_CN): δ (ppm) = 156.2, 156.1, 151.9, 151.3, 142.4, 140.6, 137.8, 137.1, 132.4, 131.7, 131.1, 128.8, 128.0, 127.7, 126.6, 126.5, 126.4, 125.4, 125.2, 125.1, 124.9, 124.0, 123.8, 123.4, 123.1, 122.0, 119, 8, 119.2; UV/vis [λ_max_ (nm) (ε (× 10^4^ M^-1^.cm^-1^)), in acetonitrile]: 238 (8.8), 281 (8.6), 347 (5.0); Luminescence [λ_max_ (nm), in dichloromethane, (77K)]: 399, (377, 388, 398).

### Synthesis of *Zn(TpyPhPyrene_2_)_2_ 2 PF_6_*

In a 50 mL Schlenk flask, **TpyPhPyrene_2_** (50 mg, 0.070 mmol) and ZnCl_2_ (5 mg, 0.037 mmol) were suspended in methanol (15 mL) and refluxed for 4 hours. Then, the solution was cooled down, filtered and the isolated material dissolved in a solution of THF (20 mL). A water solution of ammonium hexafluorophosphate was added precipitating the corresponding zinc hexafluorophosphate salt. The precipitate was filtered, washed extensively with water and ether yielding a yellow solid. Yield: 76 %, m = 47 mg (M = 1775.03 g/mol); ESI-MS: 742.30 (- 2 PF_6_); ^1^H-NMR (300 MHz, CD_2_Cl_2_): δ (ppm) = 9.00 (s, 2 H), 8.53 (m, 4 H), 8.38 (dd, ^3^J= 8.1 Hz, 2 H), 8.30-8.00 (m, 19 H), 7.89 (d, ^3^*J*= 4.8 Hz, 2 H), 7.45 (dd, ^3^J= 5.4 Hz, 2 H); UV/vis [λ_max_ (nm) (ε (× 10^4^ M^-1^.cm^-1^)), in acetonitrile]: 240 (17.2), 281 (15.6), 342 (11.1); Luminescence [λ_max_ (nm), in dichloromethane, (77K)]: 540, (377, 388, 398).

### Cyclic voltammetry

Cyclic voltammetry was performed in a three-electrode cell equipped with a platinum milli-electrode and a platinum wire counter-electrode. A silver wire served as quasi-reference electrode and its potential was checked against the ferricinium/ ferrocene couple (Fc^+^/Fc) before and after each experiment. The electrolytic media involved THF (HPLC grade), and 0.1 M of tetrabutylammonium hexafluorophosphate (TBAHP - puriss quality). All experiments were performed in a glove box containing dry, oxygen-free (< 1ppm) argon, at room temperature. Electrochemical experiments were carried out with an EGG PAR 273A potentiostat.

### Spectra

Electronic absorption spectra were recorded with a Lambda 19 NIR model from Perkin-Elmer. Fluorescence spectra were recorded in non-deoxygenated solvents (spectroscopic grade) at 20°C with a QM-4/QuantaMaster™ fluorometer from PTI® equipped with rapid mono-channel detection and continuous excitation source. Quantum yields were determined using Quinine bisulfate as a standard reference (Φ_f_ = 0.54 at 20°C in 1 N H_2_SO_4_). For time resolved spectroscopy, samples were dissolved in spectroscopic solvents and filtered (0.4 μm PVDF HPLC-filters) to remove particles and potential aggregates. The samples had an absorbance of ca. 0.7 – 0.9 (1 cm) for nanosecond transient measurements and of ca. 0.3 – 0.7 (2 mm) for femtosecond transient at the excitation wavelength. The UV/vis absorption spectra of the samples were measured before and after the laser experiments and were found to be virtually identical, thus ruling out any possible degradation or chemical change of the samples. All photophysical data reported here have a 5 to 10 % error limit, unless indicated otherwise. The experiments were performed at room temperature.

Sub-nanosecond transient absorption experiments were performed with a Spectra-Physics Hurricane Titanium:Sapphire regenerative amplifier system. The full spectrum setup was based on an optical parametric amplifier (Spectra-Physics OPA 800C) as the pump. The residual fundamental light, from the pump OPA, was used for white light generation, which was detected with a CCD spectrograph (Ocean Optics). The polarization of the pump light was controlled by a Berek Polarization Compensator (New Focus). The Berek-Polarizer was always included in the setup to provide the Magic-Angle conditions. The probe light was double-passed over a delay line (Physik Instrumente, M-531DD) that provides an experimental time window of 3.6 ns with a maximal resolution of 0.6 fs/step. The OPA was used to generate excitation pulses at 530 nm. The laser output was typically 3.5-5 μJ pulse^-1^ (130 fs FWHM) with a repetition rate of 1 kHz. The samples were placed into cells of 2 mm path length (Hellma) and were stirred with a downward projected PTFE shaft, using a direct drive spectro-stir (SPECTRO-CELL). This stir system was also used for the white light generation in a 2 mm water cell.

Time-resolved fluorescence measurements were performed on a picosecond single photon counting (SPC) setup. The frequency doubled (300-340 nm, 1 ps, 3.8 MHz) output of a cavity dumped DCM dye laser (Coherent model 700) pumped by a mode-locked Ar-ion laser (Coherent 486 AS Mode Locker, Coherent Innova 200 laser) was used as the excitation source. A (Hamamatsu R3809) micro channel plate photomultiplier was used as detector. The instrument response (∼17 ps FWHM) was recorded using the Raman scattering of a doubly de-ionized water sample. Time windows (4000 channels) of 5 ns (1.25 ps/channel) – 50 ns (12.5 ps/channel) could be used, enabling the measurement of lifetimes of 5 ps - 40 ns. The recorded traces were deconvoluted with the system response and fitted to an exponential function using the Igor Pro program.

In nanosecond pump-probe experiments, for excitation a (Coherent) Infinity Nd: YAG-XPO laser was used. The laser illuminated a slit of 10 × 2 mm. Perpendicular to this, the probe light provided by an EG&G (FX504) low pressure Xenon lamp, which irradiated the sample through a 1 mm pinhole. The overlap of the two beams falls within the first two millimeter of the cell, after the slit. The probe light from both the signal and the reference channels is then collected in optical fibers which are connected to an Acton SpectraPro-150 spectrograph which is coupled to a Princeton Instruments ICCD-576-G/RB-EM gated intensified CCD camera. Using a 5 ns gate this camera simultaneously records the spectrally dispersed light from both optical fibers on separate stripes of the CCD. De-aeration was performed by bubbling with Argon for 20 to 30 minutes.

## Figures and Tables

**Table 1.** Absorption data in acetonitrile solutions (molar absorption coefficient) for the three different systems containing the bis pyrene terpyridine.

Molecules	λ_max_^a^^bs^ nm (ε L.mol^-1^.cm^-1^)
TpyPhPyrene_2_	238 (86200), 281 (88400), 347 (50100)
^+^H_2_TpyPhPyrene_2_	a, 282 (78000), 346 (59500)
Zn(TpyPhPyrene_2_)_2_	240 (172400), 281 (156000), 342 (110600)

a overlaps with the trifluoroacetic acid absorption

**Table 2.** Fluorescence properties **TpyPhPyrene_2_** in different solvents (λ_max_ is the maximum emission, Φ is the aerated luminescence quantum yield and τ is the aerated luminescence lifetime) (λ_ex_= 310 nm).

**Fluorescence Properties**			

**Solvent**	**λ_max_ (nm)**	**Φ**	**τ (ns)**
Cyclohexane	390	0.17	18
Ether	390	0.12	13
THF	390	0.29	17
DCM	399	0.27	-a
Acetone^d^	400	0.17	20
Acetonitrile	422, 478	0.18	6.8, 16.0^b^
DMF	423, 474	0.25	6.1, 14.4^b^
BuCN^c^	377, 388, 398, 421 (s)	-	89

a compound decomposed,

b bi-exponential decay,

c 77 K,

d excited at 350 nm.

**Table 3.** Lifetimes and normalized amplitudes of **TpyPhPyrene_2_** (λ_ex_ = 310 nm) in nitrile solution.

**Nanosecond bi-exponential decay**						
	
	**λ_ILCT_^max^**	**Δf**^b^	**τ_1_ (ns)**	**Amplitude**^c^**τ_1_**	**τ_2_ (ns)**	**Amplitude τ_2_**
Butyronitrile	-a	0.275	12.2	0.70	6.2	0.30
Valeronitrile	-a	0.288	12.9	0.63	5.8	0.37
Propionitrile	462	0.290	12.2	0.21	7.9	0.79
Acetonitrile	478	0.306	16.0	0.14	6.8	0.86

a not measurable, overlap of ILCT with LE states

b Δf is the solvent polarity parameter.

Δf = (ε-1)/(2ε +1) - (n^2^-1)/(4n^2^ +2)

c Amplitudes correlate to the weight of the lifetimes as in I(t) = Σ a_i_ exp(-t/τ).

Lifetimes were determined at the maximum of the emission.

**Table 4.** Fluorescence properties **Zn(TpyPhPyrene_2_)_2_** in different solvents (λ_max_ is the emission maximum, Φ is the luminescence quantum yield and τ is the luminescence lifetime) (λ_ex_ = 310 nm).

	**Fluorescence Properties**						

	**Overall**	**^1^ILCT**			**Local Excited state**		

**Solvent**	**Φ (10^-2^)**	**λ_max_(em)**	**Φ (10^-2^)**	**τ (ns)**	**λ_max_(em)**	**Φ (10^-2^)**	**τ (ns)**
Dioxane	1.5	540	1.3	13	396	0.2	0.77
Chloroform	3.1	498	3	- a	390	0.1	-a
THF	2	518	1.6	14	390	0.4	1.7
DCM	1.9	540	1.5	- a	399	0.4	-a
Acetone^e^	0.3	580	0.1	8.3	400	0.2	1.9
Acetonitrile	0.15	616	0.03	3.0	399	0.12	1.0
BuCN	0.002	- b	0.0002	- b	399	0.0018	- b
MeOH	0.222	- b	0.002	- b	399	0.22	- b
BuCN^c^	- b	- d	- d	- d	378, 388, 398, 418 (s)	-b	21

a compound decomposed,

b not measured or undeterminable,

c 77K in butyronitrile glass,

d no phenomenon present in this condition,

e excited at 350 nm.

## References

[1] Wasielewski M.R. (1992). Photoinduced electron-transfer in supramolecular systems for artificial photosynthesis. Chem. Rev..

[2] Willemse R.J., Piet J.J., Warman J.M., Hartl F., Verhoeven J.W., Brouwer A.M. (2000). Stepwise versus direct long-range charge separation in molecular triads. J. Am. Chem. Soc..

[3] Birks J.B., Dyson D.J., Munro I.H. (1963). The relations between the fluorescence and absorption properties of organic molecules. Proc. Roy. Soc..

[4] Leroy-Lhez S., Fages F. (2005). Synthesis and photophysical properties of a highly fluorescent ditopic ligand based on 1,6-bis(ethynyl)pyrene as central aromatic core. Eur. J. Org. Chem..

[5] Fiebig T., Stock K., Lochbrunner S., Riedle E. (2001). Femtosecond charge transfer dynamics in artificial donor/acceptor systems: switching from adiabatic to nonadiabatic regimes by small structural changes. Chem. Phys. Lett..

[6] Fossum R.D., Fox M.A. (1997). Dual Exciplex Formation and Photoinduced Electron Transfer in Pyrene End-Labeled Polynorbornenes. J. Phys. Chem. B.

[7] Netzel T.L., Nafisi K., Headrick J., Eaton B.E. (1995). Direct Observation of Photoinduced Electron Transfer in Pyrene-Labeled dU Nucleosides and Evidence for Protonated 2′-Deoxyuridine Anion, dU(H), as a Primary Electron Transfer Product. J. Phys. Chem..

[8] Palit D.K., Sapre A.V., Mittal J.P. (1997). Picosecond studies on the electron transfer from pyrene and perylene excited singlet states to N-hexadecylpyridinium chloride. Chem. Phys. Lett..

[9] Zhang G.H., Thomas J.K. (1996). Energy Transfer via Ionic Processes in Polymer Films Irradiated by 0.4 MeV Electrons. J. Phys. Chem..

[10] Techert S., Schmatz S., Wiessner A., Staerk H. (2000). Photophysical Characteristics of Directly Linked Pyrene-Dimethylaniline Derivatives. J. Phys. Chem. A.

[11] Zhang G.H., Thomas J.K., Eremenko A., Kikteva T., Wilkinson F. (1997). Photoinduced charge-transfer reaction between pyrene and N,N′-dimethylaniline on silica gel surfaces. J. Phys. Chem. B.

[12] Wiessner A., Kuhnle W., Fiebig T., Staerk H. (1997). Intramolecular Charge Transfer (ICT) and Solvation of a Rigidly Linked Pyrene/N-Methylindolino Derivative and Related Compounds in Linear Alcohols. J. Phys. Chem. A.

[13] Chen L.X., Jager W.J.H., Gosztola D.J., Niemczyk M.P., Wasielewski M.R. (2000). Ionochromic Effects and Structures of Metalated Poly(p-phenylenevinylene) Polymers Incorporating 2,2′-Bipyridines. J. Phys. Chem. B.

[14] Le Bozec H., Renouard T. (2000). Dipolar and non-dipolar pyridine and bipyridine metal complexes for nonlinear optics. Eur. J. Inorg. Chem..

[15] Leroy S., Soujanya T., Fages F. (2001). Zinc(II)-operated intramolecular charge transfer fluorescence emission in pyrene-2,2′-bipyridine conjugated molecular rods. Tet. Lett..

[16] Leroy-Lhez S., Parker A., Lapouyade P., Belin C., Ducasse L., Oberle J., Fages F. (2004). Tunable fluorescence emission in pyrene-(2,2′-bipyridine) dyads containing phenylene-ethynylene bridges. Photochem. Photobiol. Sci..

[17] Benniston A.C., Harriman A., Lawrie D.J., Mayeux A. (2004). The photophysical properties of a pyrene-thiophene-terpyridine conjugate and of its zinc(II) and ruthenium(II) complexes. Phys. Chem. Chem. Phys..

[18] Wang X.Y., Del Guerzo A., Schmehl R.H. (2002). Preferential solvation of an ILCT excited state in bis(terpyridine-phenylene-vinylene) Zn(II) complexes. Chem. Commun..

[19] Soujanya T., Philippon A., Leroy S., Vallier M., Fages F. (2000). Tunable Photophysical Properties of Two 2,2′-Bipyridine-Substituted Pyrene Derivatives. J. Phys. Chem. A.

[20] Mutai T., Cheon J-D., Arita S., Araki K. (2001). Phenyl-substituted 2,2′:6′,2″-terpyridine as a new series of fluorescent compounds-their photophysical properties and fluorescence tuning. J. Chem. Soc.-Perkin Trans. 2.

[21] Erxleben A. (2003). Structures and properties of Zn(II) coordination polymers. Coord. Chem. Rev..

[22] Petitjean A., Khoury R.G., Kyritsakas N., Lehn J.M. (2004). Dynamic Devices. Shape Switching and Substrate Binding in Ion-Controlled Nanomechanical Molecular Tweezers. J. Am. Chem. Soc..

[23] Albano G., Balzani V., Constable E.C., Maestri M., Smith D.R. (1998). Photoinduced processes in 4′-(9-anthryl)-2,2′:6′,2″-terpyridine, its protonated forms and Zn(II), Ru(II) and Os(II) complexes. Inorg. Chim. Acta.

[24] Yu S.C., Kwok C.C., Chan W.K., Che C.M. (2003). Self-assembled electroluminescent polymers derived from terpyridine-based moieties. Adv. Mater..

[25] Leroy-Lhez S., Fages F. (2005). Polypyridine ligands with extended. pi.-conjugation: highly tunable fluorophores. C. R. Chimie..

[26] Harriman A., Khatyr A., Rostron S.A., Ziessel R. (2006). Light-active metal based molecular-scale wires. In Metal-Containing and Metallosupramolecular Polymers and Materials. ACS Symp. Ser..

[27] Patoux C., Launay J.P., Beley M., Chodorowski-Kimmes S., Collin J.P., James S., Sauvage J.P. (1998). Long-Range Electronic Coupling in Bis(cyclometalated) Ruthenium Complexes. J. Am. Chem. Soc..

[28] Miyaura N., Suzuki A. (1995). Palladium-Catalyzed Cross-Coupling Reactions of Organoboron Compounds. Chem. Rev..

[29] Suzuki A. (1999). Recent advances in the cross-coupling reactions of organoboron derivatives with organic electrophiles, 1995-1998. J. Organomet. Chem..

[30] Stroh C., Turek P., Rabu P., Ziessel R. (2001). Magnetic and electronic properties in novel terpyridine-based nitroxide complexes: strong radical-metal interaction via a pyridyl ring. Inorg. Chem..

[31] Storrier G.D., Colbran S.B., Craig D.C. (1997). Bis[4′-(4-anilino)-2,2′:6′,2″-terpyridine]transition-metal complexes: electrochemically active monomers with a range of magnetic and optical properties for assembly of metallo oligomers and macromolecules. J. Chem. Soc.-Dalton Trans..

[32] Storrier G.D., Colbran S.B., Craig D.C. (1998). Transition-metal complexes of terpyridine ligands with hydroquinone or quinone substituents. J. Chem. Soc.-Dalton Trans..

[33] Badger G.M., McKenzie H.A. (1953). Bond orders in aromatic compounds. Nature.

[34] Sessler J.L., Kubo Y., Harriman A. (1992). The photochemistry of pyrene-cytosine conjugates: modeling the carcinogenic action of aromatic hydrocarbons. J. Phys. Org. Chem..

[35] Rehm D., Weller A. (1969). Kinetics and mechanism of electron transfer in fluorescence quenching in acetonitrile. Berichte der Bunsen-Gesellschaft.

[36] Hagopian S., Singer L.A. (1985). Photophysical studies on 1-(p-aminophenyl)pyrene. Characterization of an intramolecular charge-transfer state with application to proton-transfer dynamics. J. Am. Chem. Soc..

[37] Berlman I.B. (1965). Handbook of Fluorescence Spectra of Aromatic Molecules.

[38] Wiessner A., Huttmann G., Kuhnle W., Staerk H. (1995). Electron Transfer, Solvation, and Amplified Stimulated Emission of Pyrene-DMA and Anthracene-DMA. J. Phys. Chem..

[39] Michalec J.F., Bejune S.A., Cuttell D.G., Summerton G.C., Gertenbach J.A., Field J.S., Haines R.J., McMillin D.R. (2001). Long-Lived Emissions from 4′-Substituted Pt(trpy)Cl+ Complexes Bearing Aryl Groups. Influence of Orbital Parentage. Inorg. Chem..

[40] Nakamoto K. (1960). Ultraviolet spectra and structures of 2,2′-bipyridine and 2,2′,2″-terpyridine in aqueous solution. J. Phys. Chem..

[41] Fink D.W., Ohnesorge W.E. (1970). Luminescence of 2,2′,2″-terpyridine. J. Phys. Chem..

[42] Murrell J.N., Methuen J.N. (1963). The theory of the electronic spectra of Organic Molecules.

[43] Verhoeven J.W., Dirkx I.P., De Boer T.J. (1969). Inter- and intramolecular donor-acceptor interactions. IV. Intramolecular charge transfer phenomena in substituted 1- aralkylpyridinium ions. Tetrahedron.

[44] Pasman P., Rob F., Verhoeven J.W. (1982). Intramolecular charge-transfer absorption and emission resulting from through-bond interaction in bichromophoric molecules. J. Am. Chem. Soc..

[45] van Walree C.A., Roest M.R., Schuddeboom W., Jenneskens L.W., Verhoeven J.W., Warman J., Kooijman H., Spek A.L. (1996). Comparison between SiMe2 and CMe2 Spacers as. sigma.-Bridges for Photoinduced Charge Transfer. J. Am. Chem. Soc..

[46] Abedin-Siddique Z., Ohno T., Nozaki K. (2004). Intense Fluorescence of Metal-to-Ligand Charge Transfer in [Pt(0)(binap)2] [binap = 2,2′-Bis(diphenylphosphino)-1,1′-binaphthyl]. Inorg. Chem..

[47] Barigelletti F., Flamigni L., Guardigli M., Sauvage J.-P., Collin J.-P., Sour A. (1996). Modulation of the luminescence properties of a ruthenium-terpyridine complex by protonation of a remote site. Chem. Com..

[48] Verhoeven J.W., Scherer T., Willemse R.J. (1993). Solvent effects on the structure of fluorescent exciplexes in rigidly, flexibly, and nonbridged donor-acceptor systems. Pure Appl. Chem..

[49] Weigel W., Rettig W., Dekhtyar M., Modrakowski C., Beinhoff M., Schlüter A.D. (2003). Dual Fluorescence of Phenyl and Biphenyl Substituted Pyrene Derivatives. J. Phys. Chem. A.

[50] von Seggern D., Modrakowski C., Spitz C., Schlüter A.D., Menzel R. (2004). Charge transfer initiated by optical excitation in diester substituted biphenylpyrene as a function of the solvent characterized by excited state absorption spectroscopy. Chem. Phys..

[51] Leroy-Lhez S., Belin C., D'Aleo A., Williams R.M., De Cola L., Fages F. (2003). Extending Excited-state Lifetimes by Interchromophoric Triplet-state Equilibration in a Pyrene-Ru(II)diimine Dyad System. Supramol. Chem..

[52] Byers G.W., Gross S., Henrichs P.M. (1976). Direct and sensitized photooxidation of cyanine dyes. Photochem. Photobiol..

[53] Nosaka Y., Kira A., Imamura M. (1981). Reactions of the excited singlet state of pyrene with metal ions. Intermolecular electron transfer in caffeine-solubilized aqueous solution. J. Phys. Chem..

[54] Daub J., Engl R., Kurzawa J., Miller S.E., Schneider S., Stockmann A., Wasielewski M.R. (2001). Competition between Conformational Relaxation and Intramolecular Electron Transfer within Phenothiazine-Pyrene Dyads. J. Phys. Chem. A.

[55] Dobkowski J., Rettig W., Waluk J. (2002). Intramolecular charge-transfer properties of a molecule with a large donor group: the case of 4′-(pyren-1-yl)benzonitrile. Phys. Chem. Chem. Phys..

[56] Kawai K., Takada T., Tojo S., Ichinose N., Majima T. (2001). Observation of Hole Transfer through DNA by Monitoring the Transient Absorption of Pyrene Radical Cation. J. Am. Chem. Soc..

[57] Kaletas B.K., Dobrawa R., Sautter A., Würthner F., Zimine M., De Cola L., Williams R.M. (2004). Photoinduced Electron and Energy Transfer Processes in a Bichromophoric Pyrene-Perylene Bisimide System. J. Phys. Chem. A.

[58] Coppo P., Duati M., Kozhevnikov V.N., Hofstraat J.W., De Cola L. (2005). White-light emission from an assembly comprising luminescent iridium and europium complexes. Angew. Chem. Int. Ed..

